# The correlation between primary ovarian insufficiency, sex hormones and immune cells: a two-step Mendelian randomization study

**DOI:** 10.3389/fendo.2025.1456273

**Published:** 2025-02-14

**Authors:** Tongtong Hong, Danhua Pu, Jie Wu

**Affiliations:** State Key Laboratory of Reproductive Medicine, Department of Obstetrics and Gynecology, The First Affiliated Hospital of Nanjing Medical University, Jiangsu Province Hospital, Jiangsu Women and Children Health Hospital, Nanjing, China

**Keywords:** primary ovarian insufficiency, immune cell traits, MR analysis, sex hormone, genetics

## Abstract

**Background:**

Primary ovarian insufficiency (POI), a cause of female infertility, is characterized by elevated gonadotropin levels and fluctuating estrogen reductions, accompanied by irregular menstruation, osteoporosis, cardiovascular disease, and genitourinary syndrome of menopause. Previous studies have shown an association between POI and immune cells, but the causal relationship remains unclear. Sex hormones play a crucial role in immune regulation by influencing the function and levels of immune cells, suggesting they may be key mediators between POI and immune cells.

**Methods:**

Utilizing genome-wide association studies (GWAS), we conducted a comprehensive bidirectional two-sample Mendelian randomization (MR) analysis to explore the causal relationship between 731 immune cell traits and POI. Furthermore, a two-step MR analysis was employed to examine the potential mediating effects of sex hormones between these two systems. To ensure the robustness of our findings, we performed extensive sensitivity analyses, evaluating heterogeneity and horizontal pleiotropy.

**Results:**

After FDR adjustment (P_FDR_ < 0.05), ten immune cell phenotypes were significantly correlated with the risk of POI. Among these, one immune cell phenotype was identified as a risk factor for POI (OR > 1), while the other nine immune cell phenotypes were protective factors (OR < 1). In the reverse MR analysis, POI was positively correlated with seven immunocyte phenotypes (OR > 1) and negatively correlated with eleven immunocyte phenotypes (OR < 1). No potential mediating effects of ten sex hormones were found between POI and immune cell traits.

**Conclusions:**

Our study comprehensively assessed the correlation between immune cell phenotypes and POI in the European population, excluding the mediating role of sex hormones, thus providing valuable insights into the biological mechanisms of POI and informing early prevention and treatment strategies.

## 1 Introduction

Primary ovarian insufficiency (POI) is a condition characterized by the decline in ovarian function among women before the age of 40, typically manifested by irregular menstruation (amenorrhea or oligomenorrhea), elevated follicle-stimulating hormone levels (FSH > 25 IU/L), and fluctuating decreases in estrogen levels (1). As reported in a recent meta-analysis, the global prevalence of POI was estimated at 3.5%, reaching up to 5.3% in developing nations (2). POI not only impacts women’s reproductive potential but also escalates the risks of osteoporosis, cardiovascular disease, and genitourinary syndrome of menopause, which imposes significant spiritual, psychological, and economic burdens on patients (3).

POI is a heterogeneous and multifactorial disorder, influenced by genetic, immune, environmental, and iatrogenic factors. Immune factors play a significant role in the development of POI, accounting for 4% to 30% of cases (1). Numerous studies have reported that individuals with POI exhibit autoimmune dysregulation, characterized by decreased Treg cells and increased activated CD4+ T lymphocytes (4, 5). A recent study reveals a reduction in the percentage of classical monocytes and NK cells, an increase in the abundance of plasma B cells, and a significantly elevated CD4/CD8 ratio in idiopathic POI patients, suggesting a close relationship between POI and immune cells (6). It is well-established that sex hormones play a crucial role in immune regulation by affecting the function and levels of immune cells. A negative correlation has been noted between plasma estradiol levels and CD8+ cell counts, while a positive correlation exists with the CD4+/CD8+ ratio (4). Positive correlations are also seen between the numbers of CD2 and CD4 T lymphocytes, B cells, and NK cells with serum LH levels, alongside negative correlations with serum FSH levels (7). Despite these findings, research on the interactions between immune cells and POI has produced conflicting results, leaving causal relationships unclear. Moreover, the potential mediating role of sex hormones between immune cells and POI has been inadequately explored.

Mendelian randomization (MR) serves as a powerful and efficient statistical approach to infer causal relationships between exposures and outcomes by leveraging genetic variants as instrumental variables (IVs). Since genetic variations are randomly assigned during gamete formation and precede disease onset, this method minimizes biases arising from confounding factors and reverse causation, thereby enhancing the reliability of causal assessments (8).

This study conducted a bidirectional MR analysis to explore the relationship between immune cells and POI. Additionally, a two-step MR analysis was performed to investigate the mediating roles of sex hormones. Our study identified several relationships previously supported by functional research and revealed many new associations lacking prior evidence. Understanding the interaction between POI and immune cells provides valuable insights for future functional studies.

## 2 Materials and methods

### 2.1 Study design

MR employs genetic instrumental variables (IVs) to determine whether a risk factor causally affects a health outcome (9). Effective IVs for causal inference must satisfy three crucial assumptions: (1) The IVs must be directly associated with the exposure; (2) The IVs must be unrelated to any confounders between the exposure and the outcome; (3) The IVs must affect the outcome solely through the exposure.

**Figure 1:**
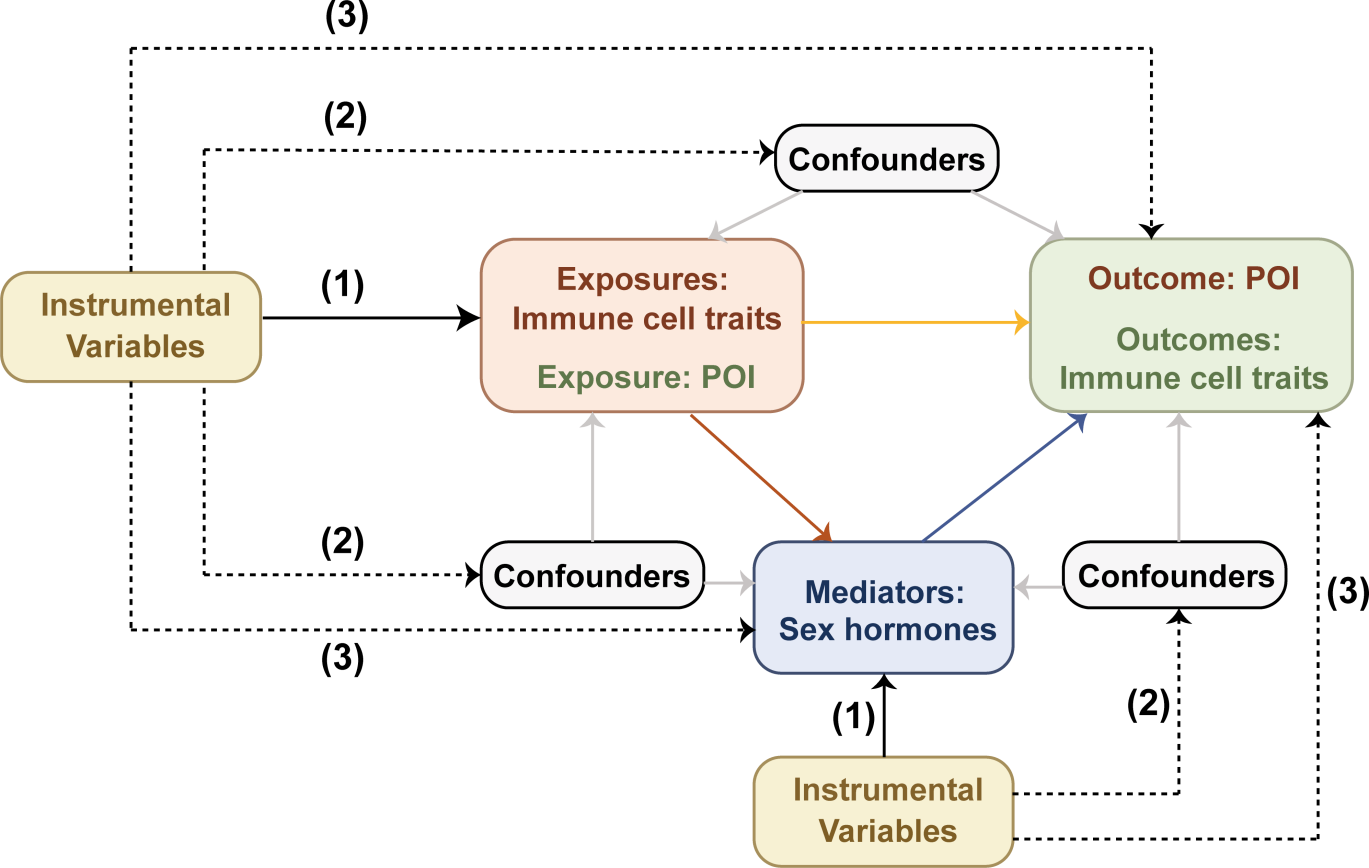
Mendelian randomization design diagram. Effective IVs for causal inference must satisfy three crucial assumptions: (1) the IVs must be directly associated with the exposure; (2) the IVs must be unrelated to any confounders between the exposure and the outcome; (3) the IVs must affect the outcome solely through the exposure. The black dashed lines denote irrelevance, while the solid black lines indicate relevance. The solid gray lines represent the effects of confounders on exposures, outcomes and mediators. The solid red, blue, and yellow lines respectively represent the causal effects of exposures on mediators, mediators on outcomes, and exposures on outcomes.

Using GWAS data, we performed a bidirectional two-sample MR analysis to investigate the correlation between 731 immune cell traits and POI, and obtained the effect size β. Additionally, we explored the potential mediating role of sex hormones between these two systems through a two-step MR analysis. First, we evaluated the causal effects of immune cell traits or POI on each mediator to derive the effect size β1. Subsequently, we assessed the causal impact of possible mediators on immune cell traits or POI to obtain the effect size β2. Finally, mediation effects were quantified using the product method, with mediation proportions calculated as (β1×β2)/β (10). Results were presented as odds ratios (ORs) with 95% confidence intervals (CIs) per standard deviation, and β values were used to calculate the mediation proportions. The research framework is illustrated in **Figure 1** (11).

### 2.2 Data sources

The GWAS data on immune cell traits were available in the GWAS catalog under accession numbers GCST90001391 to GCST90002121 (**Table 1**) (12). This study includes 731 immune cell phenotypes categorized into four groups: absolute cell counts (AC, n=118), relative cell counts (RC, n=192), median fluorescence intensity (MFI, n=389), and morphological parameters (MP, n=32). These groups encompass B cells, classical dendritic cells, mature T cells, monocytes, myeloid cells, TBNK lymphocytes, and regulatory T cells (Treg). The original GWAS analyzed 3,757 European Sardinian individuals without cohort overlap. Following adjustments for covariates (sex, age, and age^2^), correlation analyses were performed on about 22 million single nucleotide polymorphisms (SNPs) using a Sardinian sequence-based reference panel (13).

**Table 1:** Detailed information on the data sources included in this Mendelian randomization study.

Phenotypes	Data source	Phenotypic code	Sample size	Ancestry
731 immune cell traits	GWAS catalog	GCST90001391-GCST90002121	3757	European
POI	FinnGene	E4_OVARFAIL	542/218970	European
AMH	GWAS catalog	GCST90104596	7049	European
ER	IEU	prot-a-991	3301	European
SULT1E1	IEU	prot-a-2892	3301	European
FSH	GWAS catalog	GCST90012661	3484	European
LH	IEU	prot-a-529	3301	European
Oestrogen	UKBB	20003_1140884622	337159	European
Oestradiol	UKBB	30800	77633	European
SHBG	IEU	ieu-b-4870	214989	European
BTL	GWAS catalog	GCST90012102	188507	European
TTL	GWAS catalog	GCST90012112	230454	European

The GWAS data for POI is sourced from the FinnGen database (https://r10.finngen.fi/) using the data code E4_OVARFAIL. The GWAS statistics included 21,297,866 locus variations from 542 cases and 218,970 controls.

Summary data for ten sex hormones were obtained through the GWAS catalog, IEU (https://gwas.mrcieu.ac.uk/), and UKBB (https://pheweb.org/UKB-Neale/) databases. These hormones included Anti-Müllerian hormone (AMH), estrogen receptor (ER), estrogen sulfotransferase (SULT1E1), follicle-stimulating hormone (FSH), luteinizing hormone (LH), oestradiol, oestrogen, sex hormone-binding globulin (SHBG), total testosterone levels (TTL) and bioavailable testosterone levels (BTL). **Table 1** provides detailed information on all GWAS summary data.

### 2.3 Instrument variables (IVs) selection

The selection of IVs followed these criteria: First, we selected SNPs demonstrating a significant association with the exposure (p < 5 × 10^-8^ for immune cell traits and sex hormone factors; p < 1 × 10^-5^ for POI). Second, linkage disequilibrium (LD) analysis was conducted with parameters set to r² < 0.001 and a window of 10,000 kb. Third, we excluded SNPs that were significantly associated (p < 5 × 10^-8^) with the outcome (11). Finally, the F statistic was calculated for each IV to evaluate their strength and prevent weak instrument bias. The F statistic greater than 10 indicated a strong association, whereas the F statistic less than 10 indicated insufficient strength, warranting exclusion from subsequent analyses (14).

### 2.4 MR analysis

A bidirectional MR analysis was conducted to explore the association between immune cell traits and POI. Additionally, a two-step MR analysis was conducted to determine whether sex hormones mediate the relationship between immune cell phenotypes and POI.

The primary analysis used either the random-effects or fixed-effects inverse variance weighted (IVW) method (15). To mitigate false positives in multiple testing, we applied a false discovery rate (FDR) correction to associations showing nominal significance between immune cell traits and POI (P_FDR_ < 0.05 was considered statistically significant) (13). Heterogeneity among the studies was assessed using Cochran’s Q test and the associated p-values. If heterogeneity was detected (null hypothesis rejected), the random-effects IVW method was employed (15, 16). MR-Egger regression analysis was conducted to identify potential horizontal pleiotropy by assessing the p-value of the intercept (17). The MR-PRESSO (MR pleiotropy residual sum and outlier) test was also employed to identify and correct outliers in IVs, thereby reducing the influence of horizontal pleiotropy on causal estimation (18). Additionally, weighted median and simple median analyses were performed to verify the reliability and consistency of the findings (19).

### 2.5 Statistical analysis

MR analyses were conducted using R programming (version 4.1.2). The “Two Sample MR” and “MRPRESSO” packages were employed to estimate causal effects and identify outliers.

## 3 Results

### 3.1 Exploration of the causal effect of immunophenotypes on POI

#### 3.1.1 Bidirectional two-sample MR analysis

**Figure 2:**
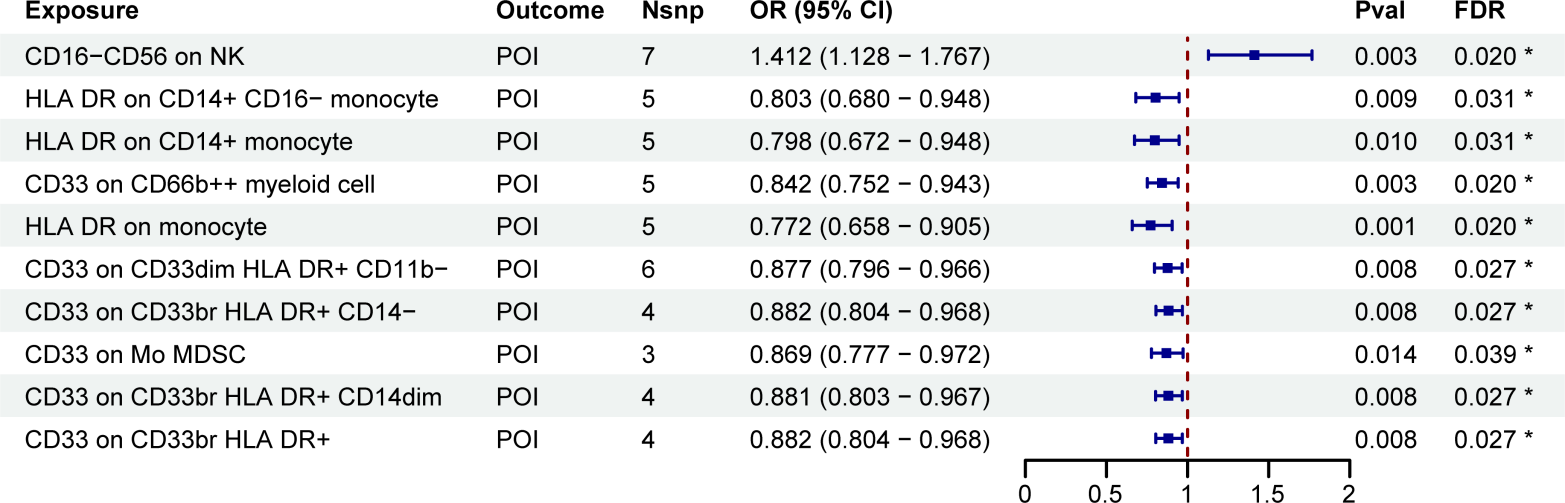
MR estimates derived from the IVW method were used to assess the effect of immune cell traits on POI. IVW, inverse variance weighting; CI, confidence interval; Nsnp, the number of single nucleotide polymorphisms; *P_FDR_<0.05.

To investigate the relationship between immune cell phenotypes and POI, we conducted a two-sample MR analysis using the IVW method as the primary approach. After adjusting for FDR with P_FDR_<0.05, we identified ten immune phenotypes significantly associated with POI. Of these, one was in the TBNK group, three were in the monocyte group, and six were in the myeloid cell group (**Supplementary Table S1**; **Figure 2**). One of the immune cell phenotypes, CD16-CD56 on NK, was positively correlated with increased POI risk (OR: 1.412, 95% CI: 1.128-1.767, P_FDR_: 0.020). Conversely, the other nine immune cell phenotypes were negatively correlated with POI risk, considered as protective factors, including HLA DR on CD14+ CD16- monocyte, HLA DR on CD14+ monocyte, CD33 on CD66b++ myeloid cell, HLA DR on monocyte, CD33 on CD33dim HLA DR+ CD11b-, CD33 on CD33br HLA DR+ CD14-, CD33 on Mo MDSC, CD33 on CD33br HLA DR+ CD14dim, and CD33 on CD33br HLA DR+ (**Supplementary Table S1**; **Figure 2**).

Furthermore, sensitivity analyses, including Cochran’s Q test, MR-Egger intercept test, and MR-PRESSO global test, produced P-values greater than 0.05, suggesting no significant heterogeneity or pleiotropy in the causal effect analysis between immune cell phenotypes and POI (**Table 2**).

**Table 2:** Heterogeneity and horizontal pleiotropy of positive results of MR analysis with immune cells as exposure and POI as outcome.

Exposure	Outcome	Heterogeneity (Cochran’s Q)	Horizontal Pleiotropy	
		P_IVW_	P_MR-PRESSO global_	P_MR-Egger intercept_
CD16-CD56 on NK	POI	0.518	0.224	0.428
HLA DR on CD14+ CD16- monocyte	POI	0.246	0.391	0.264
HLA DR on CD14+ monocyte	POI	0.241	0.386	0.267
CD33 on CD66b++ myeloid cell	POI	0.469	0.710	0.617
HLA DR on monocyte	POI	0.376	0.471	0.403
CD33 on CD33dim HLA DR+ CD11b-	POI	0.718	0.809	0.897
CD33 on CD33br HLA DR+ CD14-	POI	0.470	0.728	0.943
CD33 on Mo MDSC	POI	0.283	0.597	0.845
CD33 on CD33br HLA DR+ CD14dim	POI	0.475	0.723	0.937
CD33 on CD33br HLA DR+	POI	0.474	0.727	0.946

#### 3.1.2 Two-step MR analysis

**Figure 3:**
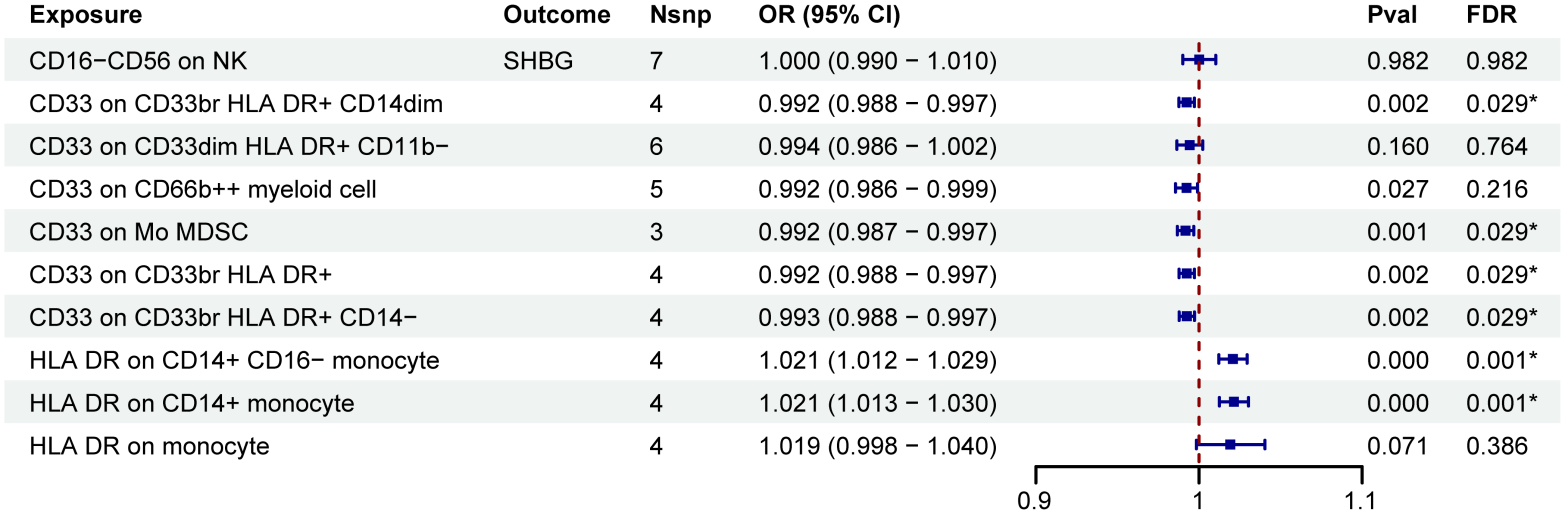
MR estimates derived from the IVW method were used to assess the effect of 10 immune cell traits on SHBG. IVW, inverse variance weighting; CI, confidence interval; Nsnp, the number of single nucleotide polymorphisms; *P_FDR_<0.05.

**Figure 4:**
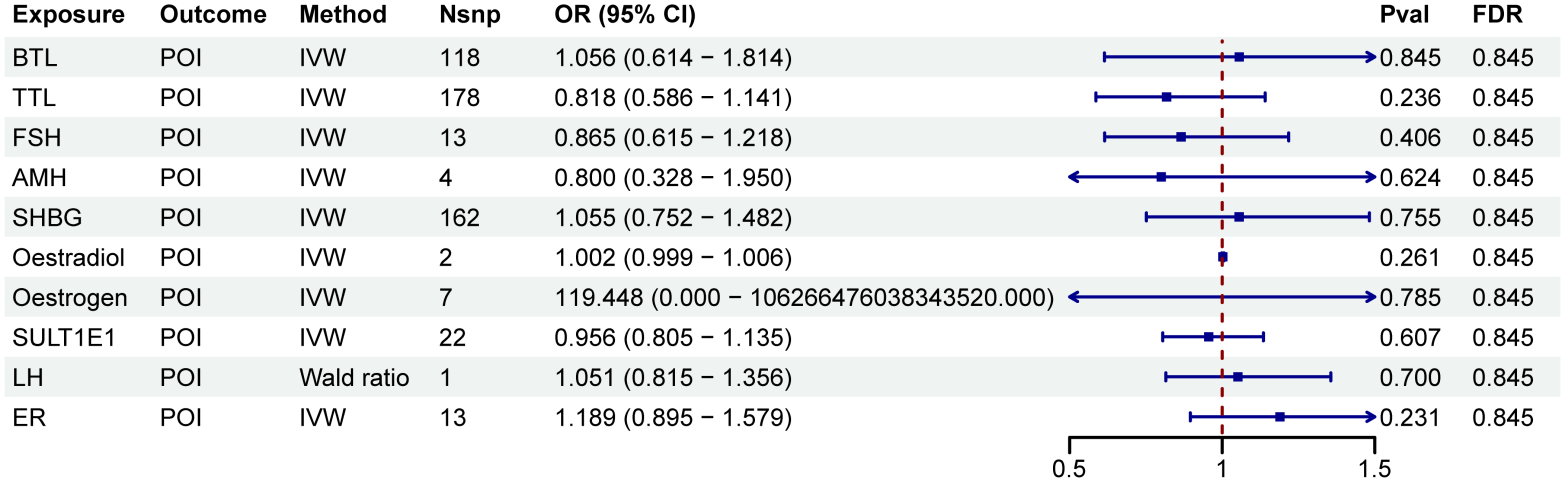
Forest plots illustrated the effect of sex hormones on POI. IVW, inverse variance weighting; CI, confidence interval; Nsnp, the number of single nucleotide polymorphisms.

A two-step MR analysis was performed to investigate the potential mediating effects of sex hormones, including AMH, ER, SULT1E1, FSH, LH, oestradiol, oestrogen, SHBG, TTL and BTL. Initially, we investigated the relationship between ten immune cell phenotypes significantly associated with POI and the sex hormones (**Supplementary Figure 1**; **Supplementary Table S2**). After FDR adjustment, most immune cell phenotypes showed no significant associations with sex hormones (P_FDR_>0.05). Only CD33 on CD33br HLA DR+ CD14dim, CD33 on Mo MDSC, CD33 on CD33br HLA DR+, CD33 on CD33br HLA DR+ CD14−, HLA DR on CD14+ monocyte and HLA DR on CD14+ CD16− monocyte exhibited significant associations with SHBG (P_FDR_<0.05, **Figure 3**; **Supplementary Figure 1**; **Supplementary Table S2**). However, no correlation was found between SHBG and POI (P_FDR_>0.05, **Figure 4**; **Supplementary Table S3**).

### 3.2 Exploration of the causal effect of POI on immunophenotypes

#### 3.2.1 Bidirectional two-sample MR analyses

**Figure 5:**
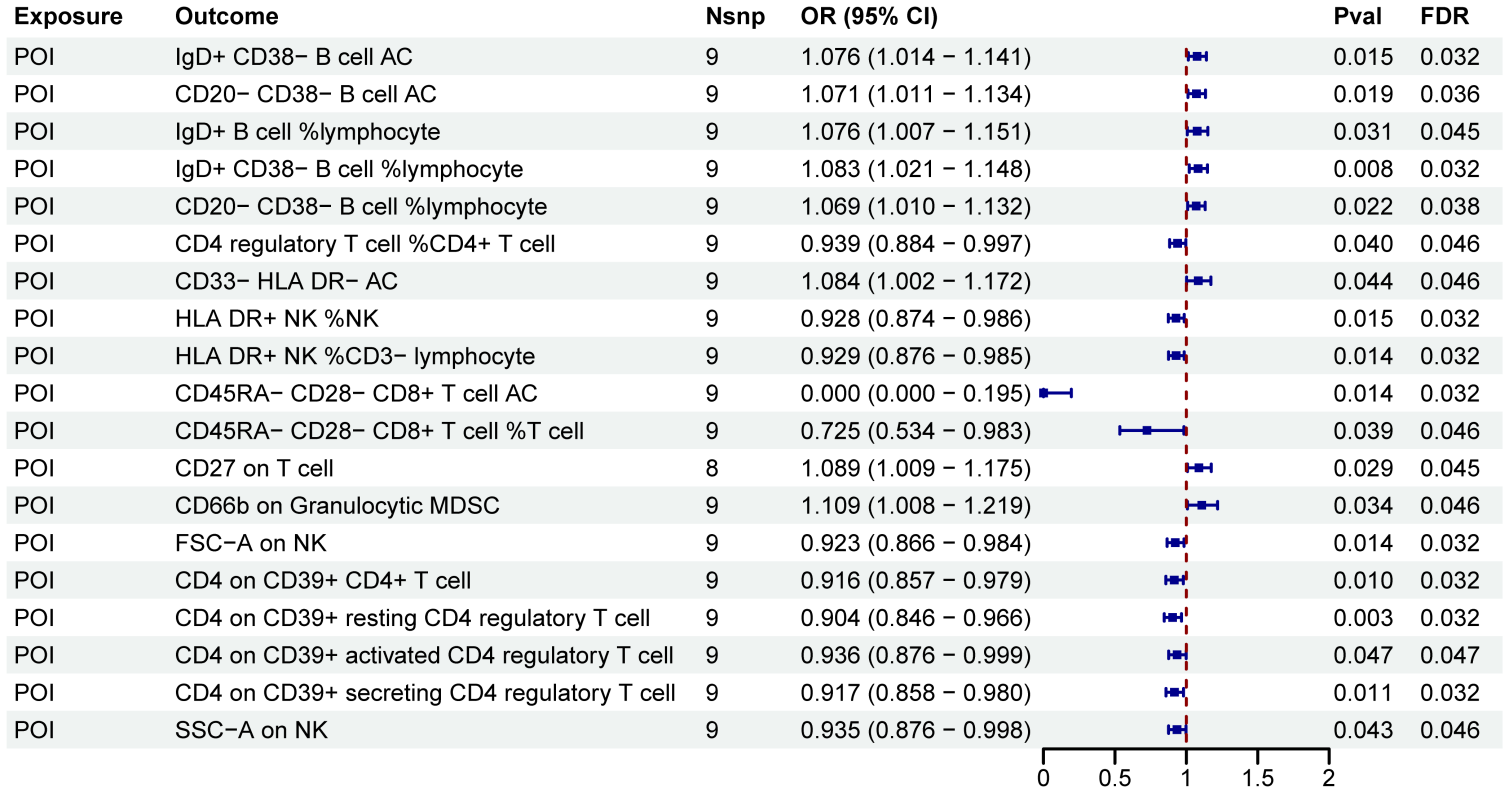
MR estimates derived from the IVW method were used to assess the effect of POI on immune cell traits. IVW, inverse variance weighting; CI, confidence interval; Nsnp, the number of single nucleotide polymorphisms.

After FDR adjustment (P_FDR_<0.05), we detected significant effects of POI on nineteen immune cell phenotypes (**Figure 5**; **Supplementary Table S4**). Further sensitivity analysis excluded phenotypes potentially influenced by heterogeneity or pleiotropy bias, and CD27 on T cell did not pass the pleiotropy test (**Table 3**). Ultimately, POI was associated with eighteen immune cell traits, which primarily belonged to the B cell, Treg, TBNK, and myeloid cell panels. We found that POI onset could increase the levels of seven immune cell phenotypes (OR>1): IgD+ CD38− B cell AC, CD20− CD38− B cell AC, IgD+ B cell %lymphocyte, IgD+ CD38− B cell %lymphocyte, CD20− CD38− B cell %lymphocyte, CD33− HLA DR− AC and CD66b on Granulocytic MDSC (**Figure 5**; **Supplementary Table S4**). Additionally, eleven immune cell phenotypes were found to be decreased in POI patients (OR<1): CD4 regulatory T cell %CD4+ T cell, HLA DR+ NK %NK, HLA DR+ NK %CD3− lymphocyte, CD45RA− CD28− CD8+ T cell AC, CD45RA− CD28− CD8+ T cell %T cell, FSC−A on NK, CD4 on CD39+ CD4+ T cell, CD4 on CD39+ resting CD4 regulatory T cell, CD4 on CD39+ activated CD4 regulatory T cell, CD4 on CD39+ secreting CD4 regulatory T cell, SSC−A on NK (**Figure 5**; **Supplementary Table S4**).

**Table 3:** Heterogeneity and horizontal pleiotropy of positive results of MR analysis with POI as exposure and immune cells as outcome.

Exposure	Outcome	Heterogeneity (Cochran’s Q)	Horizontal Pleiotropy	
		P_IVW_	P_MR-PRESSO global_	P_MR-Egger intercept_
POI	IgD+ CD38- B cell AC	0.632	0.117	0.301
POI	CD20- CD38- B cell AC	0.944	0.895	0.703
POI	IgD+ B cell %lymphocyte	0.213	0.155	0.324
POI	IgD+ CD38- B cell %lymphocyte	0.879	0.088	0.542
POI	CD20- CD38- B cell %lymphocyte	0.985	0.958	0.954
POI	CD4 regulatory T cell %CD4+ T cell	0.585	0.236	0.350
POI	CD33- HLA DR- AC	0.442	0.587	0.474
POI	HLA DR+ NK %NK	0.840	0.543	0.979
POI	HLA DR+ NK %CD3- lymphocyte	0.816	0.799	0.735
POI	CD45RA- CD28- CD8+ T cell AC	0.573	0.414	0.691
POI	CD45RA- CD28- CD8+ T cell %T cell	0.380	0.194	0.284
POI	CD27 on T cell	0.559	0.026	0.180
POI	CD66b on Granulocytic MDSC	0.551	0.709	0.928
POI	FSC-A on NK	0.596	0.394	0.602
POI	CD4 on CD39+ CD4+ T cell	0.566	0.267	0.785
POI	CD4 on CD39+ resting CD4 regulatory T cell	0.759	0.395	0.180
POI	CD4 on CD39+ activated CD4 regulatory T cell	0.463	0.430	0.168
POI	CD4 on CD39+ secreting CD4 regulatory T cell	0.402	0.080	0.196
POI	SSC-A on NK	0.444	0.449	0.097

#### 3.2.2 Two-step MR analysis

**Figure 6:**
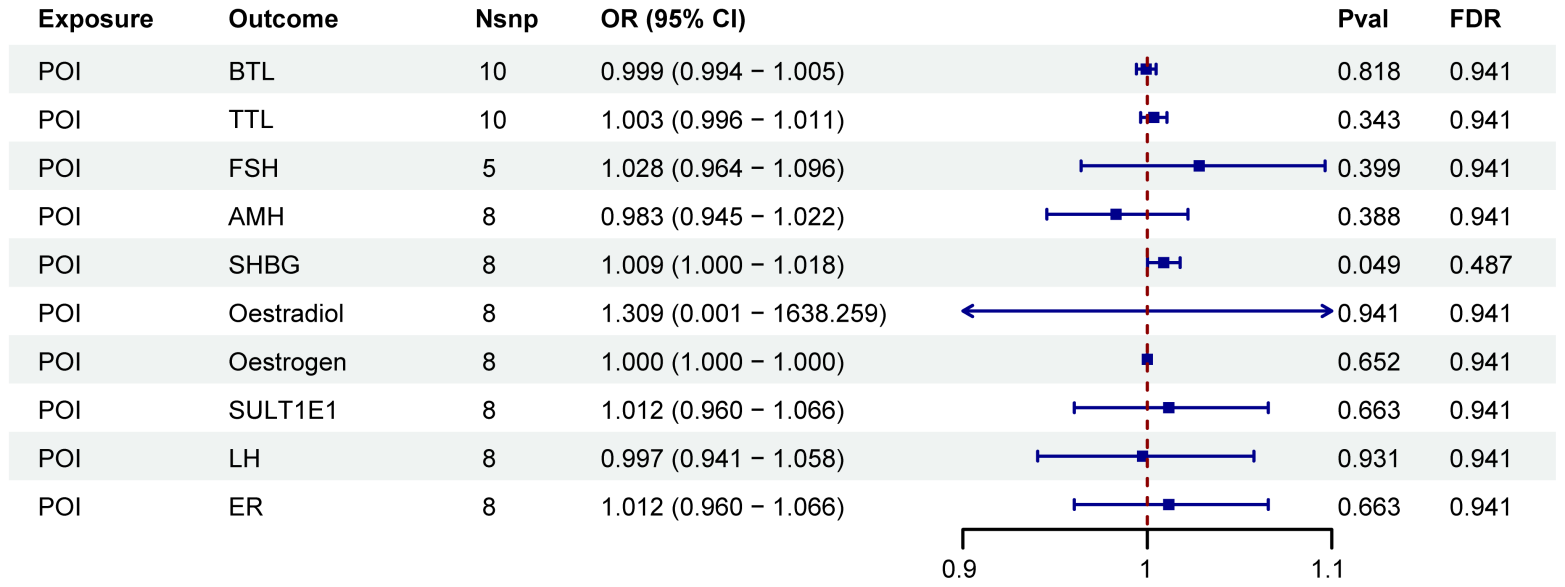
Forest plots illustrated the effect of POI on mediators. IVW, inverse variance weighting; CI, confidence interval; Nsnp, the number of single nucleotide polymorphisms.

As shown in **Figure 6**, POI was associated with SHBG (P < 0.05). However, after FDR adjustment, no significant effects of POI on SHBG were detected (**Figure 6**; **Supplementary Table S5**). Additionally, no correlation was found between SHBG and the nineteen immune cell phenotypes associated with POI (**Supplementary Figure 2**).

## 4 Discussion

Using a bidirectional two-sample and two-step MR study, we investigated the relationships and potential mediating effects of sex hormones between immune cell traits and POI. Our analysis revealed that out of 731 immune cell phenotypes studied, POI exerted significant effects on 18 immune phenotypes, notably B cells, Treg cells, and TBNK lymphocytes (P_FDR_ < 0.05). Furthermore, 10 immune phenotypes, primarily monocytes and myeloid cells, demonstrated significant effects on POI (P_FDR_ < 0.05). However, serum sex hormones did not mediate these relationships.

Monocytes are essential to the immune system, participating in inflammatory responses and tissue repair (20). Our analysis revealed a correlation between elevated levels of monocytes (HLA DR on CD14+ CD16- monocyte, HLA DR on CD14+ monocyte, and HLA DR on monocyte, OR<1) and a reduced risk of POI, which is consistent with recent studies reporting a decrease in CD14+ monocytes in POI patients (6). Additionally, we identified that myeloid cells (including CD33 on CD66b++ myeloid cell, CD33 on CD33dim HLA DR+ CD11b-, CD33 on CD33br HLA DR+ CD14-, CD33 on Mo MDSC, CD33 on CD33br HLA DR+ CD14dim, and CD33 on CD33br HLA DR+, OR<1) acted as protective factors against POI (21). Myeloid cells are integral to both innate and adaptive immune responses, as well as tissue repair and regeneration (21). Therefore, they may influence the onset of POI by modulating the local immune environment, inflammatory responses, and tissue repair. However, the role of myeloid cells in the development of POI remains underexplored in both clinical and basic research, offering a direction for future studies.

Natural Killer (NK) cells are crucial immune system components, contributing to immune regulation through direct cytotoxic effects and cytokine secretion (22). Elevated NK cell levels in the ovaries have been reported to disrupt normal follicular development by exerting cytotoxic effects on granulosa cells, which play a crucial role in follicular growth and maturation (23). Additionally, some studies have demonstrated abnormally low NK cell activity in POI patients (24–26). This study revealed that elevated levels of CD16-CD56 on NK were associated with an increased risk of POI. Furthermore, POI onset could reduce levels of specific NK cells, including HLA-DR+ NK %NK, FSC-A on NK, and SSC-A on NK. Our findings provided additional insights into the relationship between NK cell subtypes and POI.

Existing research on the relationship between B cells and POI remains controversial. While some studies have reported significantly higher B cell counts in POI patients (27), others, such as Mignot et al., found no significant difference in B cell counts between POI patients and healthy controls (28). B cells, the second-largest group of lymphocytes, are vital for several immune functions, including antigen presentation, cytokine secretion, and antibody production (6). Our study demonstrated that POI onset could increase B cell levels.

T cells are categorized into three subgroups: helper T cells, cytotoxic T cells, and Treg cells. Treg cells are essential for immune regulation, maintaining homeostasis, and preventing autoimmunity (29). Our research showed that POI led to a reduction in Treg cell levels. Recent studies suggest that POI patients exhibit abnormal cellular immunity in both the peripheral blood and ovarian microenvironments, characterized by decreased Treg cell numbers and impaired immunosuppressive function, which is consistent with our results (30, 31). Lei et al. found through an MR study that higher proportions of activated and secreting Treg cells were associated with osteoporosis prevention (13). Thus, the increased susceptibility to osteoporosis in POI may be due to the decline in Treg cell levels.

POI is characterized by low estrogen and high gonadotropin levels. Estrogen is known to participate in the proliferation and activation of T cells and B cells while regulating the anti-inflammatory functions of Treg cells (30, 32–37). Based on this, we hypothesized that POI might lead to reduced estrogen levels, subsequently resulting in decreased Treg cell levels. However, our study did not find evidence that serum sex hormones mediated the relationship between POI and immune cell phenotypes, which suggested that the changes in immune cell dynamics in POI may be direct effects of the condition itself rather than secondary to hormonal alterations. Nevertheless, MR is merely an analytical method based on IVs, and its negative results cannot definitively rule out the presence of mediating factors.

There are several strengths in our study. Firstly, through MR analysis, we offered a novel perspective on the relationship between immune cell traits and POI. Secondly, utilizing large-scale GWAS cohorts ensures robust statistical efficiency. Thirdly, our conclusions are based on genetic instrumental variables, which effectively minimize biases from reverse causation and confounders commonly present in traditional observational studies. Lu et al. conducted a study similar to ours, focusing on the causal effect of immune cells on POI (38). Unlike their study, we further examined reverse causality, specifically how POI affected immune cells while accounting for the mediating role of sex hormones. Although sex hormones were not identified as mediators, our study provided a more comprehensive understanding of the relationship between POI and immune cells. Understanding the pathogenesis of POI can facilitate its prevention by detecting related immune cell phenotypes. Additionally, targeting these immune cell phenotypes altered in POI could be a viable strategy for managing the clinical symptoms of POI, offering a new treatment approach for patients who cannot undergo hormone replacement therapy. In an autoimmune mouse model of POI, Treg cells were observed to preserve ovarian function by inhibiting ovarian cell apoptosis through the Akt/FOXO3a signaling pathway (31). Furthermore, osteoporosis in ovariectomized rats was improved by adjusting the Th17/Treg balance, underscoring the therapeutic potential of modulating immune cells to manage POI (39).

However, our study has limitations as well. Although multiple sensitivity analyses were conducted to minimize bias, it is unlikely that all biases could be fully eliminated. As MR is primarily a method for analyzing relationships between exposure and outcome and may not fully capture the complexity of immune responses in individuals, further studies are needed to confirm the relationship between immune cell phenotypes and POI using *in vitro* and *in vivo* experimental models. Additionally, the GWAS data used in our analysis were predominantly sourced from the European population. Given the genetic heterogeneity among different ethnic groups, these results may not be universally applicable to other populations. As a preliminary study in understanding the potential correlation between immune cells and POI, our findings will serve as a foundation for further exploration of the relationship between POI and immune cells.

## 5 Conclusions

In summary, our comprehensive bidirectional two-sample two-step MR analysis indicated a correlation between immune cell phenotypes and POI, suggesting the interplay between these two systems and excluding the mediating role of sex hormones in the European population. This finding may facilitate the development of biomarkers for the early detection and prognosis of POI, as well as inform the design of targeted therapeutic interventions. However, due to the limitations in MR analysis, further basic research and clinical trials are necessary to confirm the association between immune cells and POI.

## Data Availability

The original contributions presented in the study are included in the article/**Supplementary Material**. Further inquiries can be directed to the corresponding author.
