# Supplementary material for: The correlation between primary ovarian insufficiency, sex hormones and immune cells: a two-step Mendelian randomization study
Source: Front Endocrinol (Lausanne). 2025 Feb 14;16:1456273. doi: 10.3389/fendo.2025.1456273 (PMC11868816; doi:10.3389/fendo.2025.1456273)
Supplement: Supplementary file 1 [file DataSheet1.zip › Supplementary Material/Supplementary Figure/Supplementary Figure.docx]

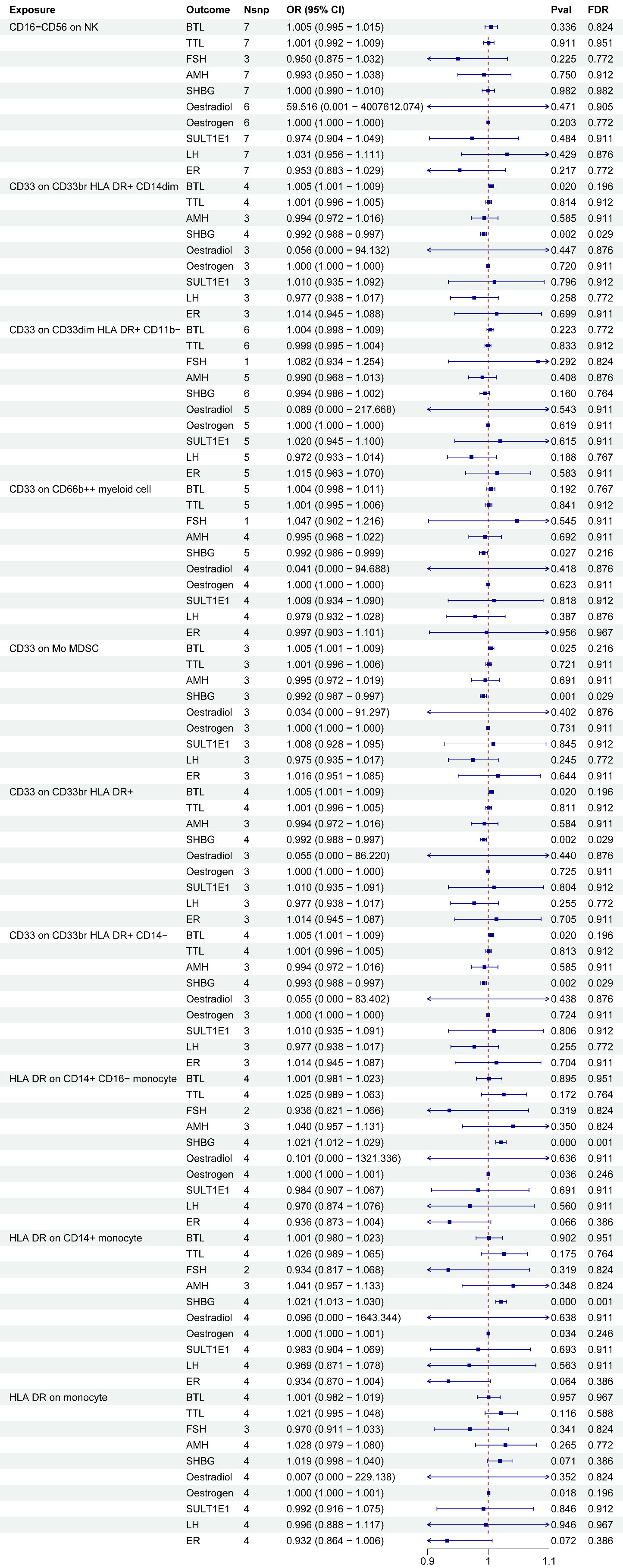


**Supplementary Figure 1.** Forest plots illustrated the effect of 10 immune cell traits on sex hormones. IVW: inverse variance weighting; CI: confidence interval; Nsnp: the number of single nucleotide polymorphisms.


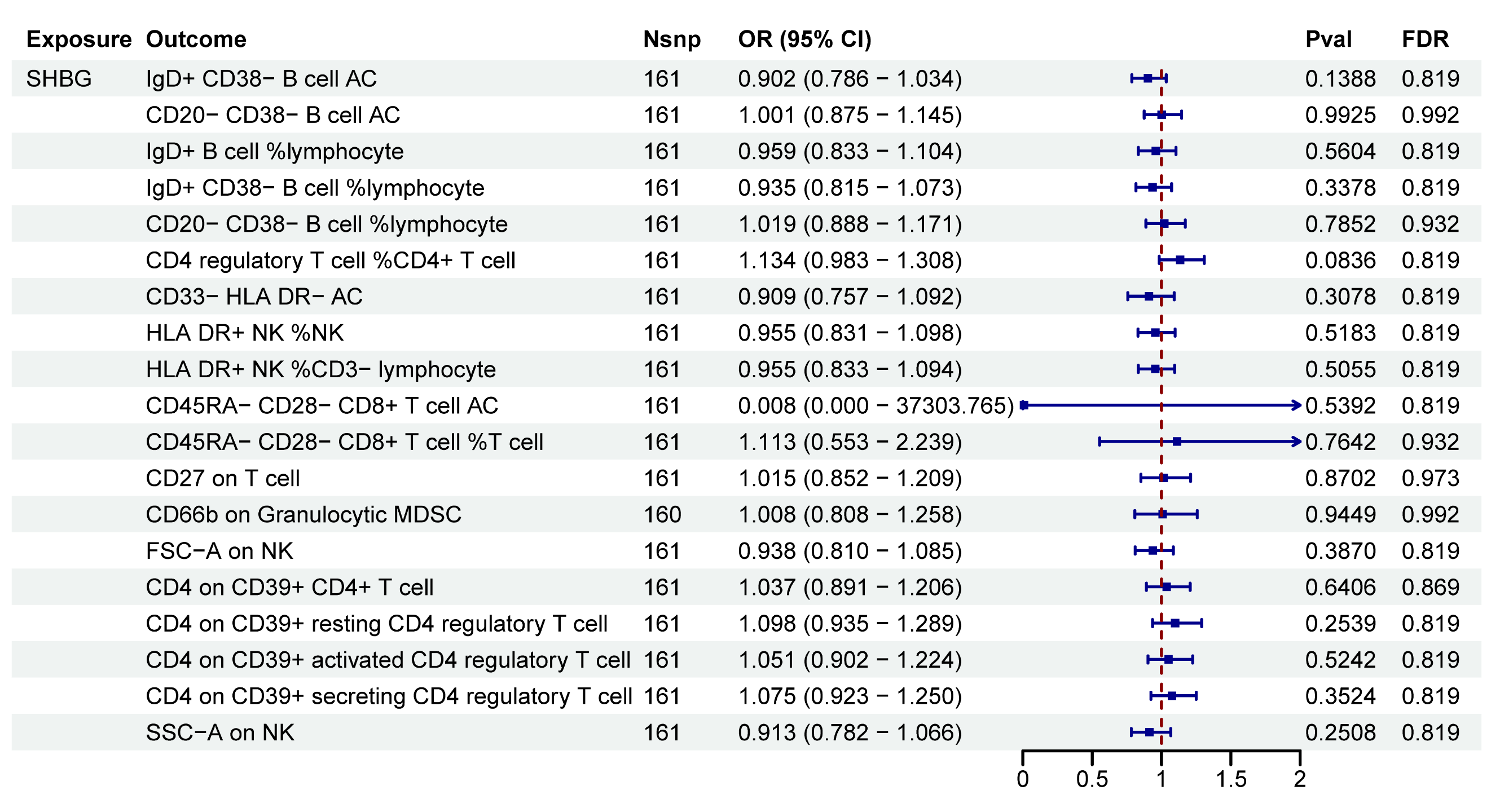


**Supplementary Figure 2.** MR estimates derived from the IVW method were used to assess the causal effect of SHBG on the 19 immune cell phenotypes associated with POI. IVW: inverse variance weighting; CI: confidence interval; Nsnp: the number of single-nucleotide polymorphism.
